# Comparison of serological assays for detecting antibodies in ducks exposed to H5 subtype avian influenza virus

**DOI:** 10.1186/1746-6148-8-117

**Published:** 2012-07-23

**Authors:** Hendra Wibawa, Joerg Henning, Dessie Eri Waluyati, Tri Bhakti Usman, Sue Lowther, John Bingham, Akhmad Junaidi, Joanne Meers

**Affiliations:** 1 CSIRO-Australian Animal Health Laboratory, Geelong, VIC, Australia; 2 School of Veterinary Science, The University of Queensland, Gatton Campus, Gatton, QLD, Australia; 3 Disease Investigation Centre Wates, Yogyakarta, Indonesia; 4 Present address: Directorate General of Livestock and Animal Health Services, Ministry of Agriculture, Jakarta, Indonesia

**Keywords:** Avian influenza, H5N1, Hemagglutination inhibition test, Virus neutralization test, Horse red blood cells, Duck, Kappa

## Abstract

**Background:**

Chicken red blood cells (RBCs) are commonly used in hemagglutination inhibition (HI) tests to measure hemagglutinating antibodies against influenza viruses. The use of horse RBCs in the HI test can reportedly increase its sensitivity when testing human sera for avian influenza antibodies. This study aims to compare the proportion of positives detected and the agreement between two HI tests using either chicken or horse red blood cells for antibody detection in sera of ducks experimentally infected or naturally exposed to Indonesian H5 subtype avian influenza virus. In addition, comparison with a virus neutralisation (VN) test was conducted with the experimental sera.

**Results:**

In the experimental study, the proportion of HI antibody-positive ducks increased slightly, from 0.57 when using chicken RBCs to 0.60 when using horse RBCs. The HI tests indicated almost perfect agreement (kappa = 0.86) when results were dichotomised (titre ≥ 4 log2), and substantial agreement (weighted kappa = 0.80) for log titres. Overall agreements between the two HI tests were greater than between either of the HI tests and the VN test. The use of horse RBCs also identified a higher proportion of antibody positives in field duck sera (0.08, compared to chicken RBCs 0.02), with also almost perfect agreements for dichotomized results (Prevalence and bias adjusted Kappa (PABAK) = 0.88) and for log titres (weighted PABAK = 0.93), respectively. Factors that might explain observed differences in the proportion of antibody-positive ducks and in the agreements between HI tests are discussed.

**Conclusion:**

In conclusion, we identified a good agreement between HI tests. However, when horse RBCs were used, a higher proportion of sera was positive (titre ≥ 4 log2) than using chicken RBCs, especially during the early response against H5N1 virus. The HRBC-HI might be more responsive in identifying early H5N1 HPAI serological response and could be a recommended assay for avian influenza sero-surveillance in both wild and domestic birds.

## Background

The spread of the Eurasian lineage of H5N1 highly pathogenic avian influenza (HPAI) from China to other countries across Asia, Europe, the Middle East, and Africa is an unprecedented epizootic event. Although the initial outbreaks of H5N1 HPAI virus in Hong Kong, China, were successfully eradicated in late 1997 [1], the virus re-emerged in 2001 and 2002 causing HPAI outbreaks with high mortalities of chickens on commercial farms [1] and deaths of migratory birds and waterfowl, including ducks, in two local parks in Hong Kong [2]. At least three waves of H5N1 HPAI spread then occurred [3]: firstly, to East Asia and Southeast Asia between 2003 and 2004 [4,5]; secondly, from Qinghai Lake, China, to South Asia, Europe, the Middle East, and Africa between 2005 and 2006 [5,6]; and thirdly, to South Asia and Southeast Asia again between 2007 and 2009 [7-10].

Given the continuing evolution and the endemicity of H5N1 HPAI viruses in many countries, and the catastrophic impacts to both poultry and human health [3], rapid and sensitive diagnostic methods are very important for early detection of H5N1 disease outbreaks. The haemagglutination inhibition (HI) test is one such method, being relatively quick to perform and widely regarded as a reliable method for the detection of antibodies to influenza viruses. The HI test relies on the inhibition of the interaction between the viral hemagglutinin (HA) glycoprotein and sialic acid receptors on the surface of red blood cells (RBCs) by antibodies which are directed against the HA receptor binding pocket [11]. This test is a simple and inexpensive technique utilizing standard laboratory equipment, and can be used for identification of avian influenza virus subtypes as well as for measuring HA specific antibodies to the virus [12]. For these reasons, the HI test has been used extensively in epidemiological studies of influenza virus [13]. Some studies [14-16] have shown that the HI tests, using various types of RBCs, were less sensitive than the virus neutralization (VN) tests in detecting the antibody response of humans who were naturally exposed to influenza viruses. In contrast, others reported that in some circumstances, the HI test using horse or goose RBCs could be more sensitive than the neutralization test [17]. These disparate findings suggest that the sensitivity of serology tests can be variable, and may depend on the particular materials or methods that are used.

The sensitivity of the HI test for detection of antibodies against avian influenza viruses in human sera can be improved by replacing avian RBCs with equine RBCs within the test [13,14,17-19]. An experimental study using sera of gallinaceous birds, pheasants and chukar partridges, revealed that HI-antibody titres that were detected by using horse RBCs were more comparable to neutralizing antibody titres than those from HI tests using chicken RBCs [20]. None of these studies has tested serum samples from aquatic birds, such as domestic ducks, which are considered to play an important role in the H5N1 HPAI virus maintenance and spread [21-23]. Since the avian influenza antibody titres of ducks are reportedly low when measured by the traditional HI test based on chicken RBCs [24,25], we hypothesised that the use of horse RBCs could influence the performance of the HI test. We tested sera from ducks experimentally inoculated and naturally exposed in the field to Indonesian H5 subtype virus using HI tests based on both chicken (HI-CRBC) and horse RBCs (HI-HRBC) and using a virus neutralisation (VN) test for sera from the experimentally infected ducks.

## Methods

### Sera

The studies were conducted on the two different groups of serum samples (experimental and field) at two different laboratories: Australian Animal Health Laboratory (AAHL), Geelong, Australia and Disease Investigation Centre (DIC) Wates, Yogyakarta, Indonesia, respectively.

Sixty serum samples were collected from experimentally infected ducks (N = 10) at six sampling times: 4 days prior to the virus inoculation and 8, 15, 22, 29 and 34 day post inoculation (dpi). The results of a blocking enzyme-linked immunosorbent assay showed that none of these ducks had pre-exposure to avian influenza (AI) virus subtypes. Each duck was inoculated via nostrils, eyes and mouth with 0.5 ml of inoculum containing 10^8.4^ 50% egg lethal doses of an Indonesian clade 2.1.1 H5N1 virus, A/duck/Sleman/BBVW-1003-34368/2007 (IDN34368), derived from infected allantoic fluid and diluted in sterile phosphate-buffered saline (PBS). The trial was approved by the CSIRO-AAHL Animal Ethics Committee. The tests on experimentally-derived sera were conducted at microbiological physical containment level 3 at AAHL.

Field sera were collected in a longitudinal study conducted to estimate HPAI prevalence in farmed duck populations [26]. In this study, 54 “moving” duck flocks in 6 districts of Central Java, Indonesia, were sampled at monthly intervals between November 2008 and April 2009 [26]. The highest bird-level seroprevalence was observed in December 2008. Therefore, December 2008 represented the month with the largest range of HI titres and serum samples collected in this month were used for the current study. Sera from a total of 518 ducks were available and were tested at the DIC Wates. From all ducks also cloacal and an oropharyngeal swabs were collected. The swab samples were then tested at the DIC Wates in pools of five for subtype H5 virus RNA using influenza RT-PCR as described in an earlier study [27]. All pools of our study birds tested H5 virus RNA negative.

### HI tests

For HI tests conducted at AAHL on experimental duck sera, antigen of the same virus isolate used in the inoculation was used in the tests. Chicken blood in EDTA was obtained from the small animal facility at AAHL and horse blood in Alseiver’s solution was obtained from the Institute of Medical and Veterinary Science, Adelaide, Australia. For HI tests conducted at DIC Wates on field-derived sera, antigen of another Indonesian 2.1.1 clade virus, A/chicken/Legok/2003 (H5N1) was used in the tests. Chicken and horse blood, both in Alseiver’s solution, were obtained from chickens housed at DIC and from PT Bio Farma, Bandung, Indonesia, respectively. Each blood sample was washed three times with 0.1 M PBS (pH 7.2-7.3) and prepared as 10% RBCs stock solution. In both studies, serum from the blood donor chickens or horses was confirmed to be H5 antibody negative before use in the HI tests.

The sera from both the experimental and field studies were heat inactivated for 1 hour at 56°C before use. It is not necessary to treat duck sera with receptor destroying enzymes, because sera from the majority of avian species do not contain nonspecific inhibitors [12,28]. However, sera from species other than chickens may sometimes cause non-specific agglutination of chicken RBCs [28] and possibly of other species RBCs. Prior adsorption of avian sera with the RBCs that are used in the HI test can remove these non-specific agglutinins [12,28,29]. Thus, prior to the tests, 50 μl of each duck serum sample was adsorbed with 50 μl of 10% chicken RBCs in PBS or with 50 μl of 10% horse RBCs in PBS containing 0.5% bovine serum albumin (BSA [Sigma Aldrich]) in 96-well U-bottom microtiter plates, incubated at 4°C for 1 hour and gently mixed periodically. Here, the sera were regarded as a 1:2 dilution of the original serum samples. After the incubation, RBCs were pelleted by centrifugation at 800 ***g*** for 5 minutes. The adsorbed sera were removed, then 25 μl was transferred to the first column wells of another microtitre plate containing 25 μl PBS (for HI-CRBC) or PBS + 0.5% BSA (for HI-HRBC). This means that these sera were further diluted 1:2 at the start of the HI test, therefore the minimal detectable titre given by the HI tests was 4 (2 log2). The HI tests were performed with 0.5% chicken RBCs in PBS or with 1% horse RBCs in PBS + 0.5% BSA using 4 haemagglutinating units (HAU) of antigen per well as described previously [14,30]. Reference positive serum (from immunized specific-pathogen free [SPF] chickens with A/chicken/Indonesia/Wates1/2005 H5N1 antigen) and negative serum (from uninfected SPF chickens) were included on each run of the test. The HI titre was expressed as the reciprocal value of the highest dilution of serum causing complete inhibition of agglutination of 4 HAU antigen. To dichotomize positive from negative results, HI titres of 16 (4 log2) or greater were classified as positive for avian influenza antibody, according to OIE guidelines [28].

### VN test

The VN test was performed on the experimentally-derived sera. Initially, 50 μl of cell culture media containing EMEM (Invitrogen) with HEPES solution, glutamine, penicillin and streptomycin, and fungizone (all antibiotics from Sigma Aldrich) was added to all wells of a 96-well microtitre plate. Sera were firstly diluted 1:2 in sterile PBS; then 50 μl of each diluted serum was added each into the wells of the first and second column of microtitre plates. Starting from the wells in the second column, two-fold dilutions of 50 μl volumes of sera were performed across the plate, then the excess of 50 μl volumes were discarded after the last wells. The same positive and negative control sera as HI tests were diluted in the same way. The virus stock (IDN34368) was diluted to contain 100 TCID_50_/50 μl, and 50 μl was added to all wells containing diluted sera, except for the first wells which served as serum controls. Plates were incubated at 37°C, in 5% CO2, for 1 hour, then 100 μl Vero cells suspension containing approximately 2–4 × 10^5^ cells/ml in culture media with 10% foetal calf serum (Invitrogen) was added to all wells of the plates and the plates were incubated at 37°C in 5% CO_2_. A back titration of diluted virus was performed with every test run to confirm the concentration of virus used in the test. The VN plates were examined for cytopathic effect (CPE) using an inverted microscope after 5 days incubation. The neutralization titre was expressed as the reciprocal value of the highest serum dilution at which viral CPE was not observed. For consistency with the HI test results, any serum with neutralizing antibody titre of 16 (4 log2) or greater was considered as positive.

### Statistical analysis

For the samples obtained in the experimental study, we calculated the proportion of positive test results for each serological test (HI-CRBC, HI-HRBC, VN) at each sampling, including the 95% binominal exact confidence intervals for each sampling period [31]. We also calculated the overall proportion of positive test results for each serological test and the 95% confidence interval. As the same birds were repeatedly tested at different sampling periods, repeated observations of birds in the overall calculation were considered by accounting for a clustering of birds by sampling period [32]. This resulted in the adjustment of standard errors and thereby adjustment of confidence intervals. For the samples collected in the field study, we calculated the proportion of positive test results for the two serological tests used (HI-CRBC, HI-HRBC) and the 95% confidence interval. Some birds were obtained from the same farms; therefore we considered a clustering of birds by duck farms and adjusted the standard errors and confidence intervals for this intra-farm correlation [32].

The agreement of dichotomised test results between two tests was assessed using the kappa (κ) statistic. As a tendency of one test identifying more positives than another test influences the kappa statistic, we used the McNemar test for paired data to evaluate the proportion of positives identified between the two tests [33]. A significant result indicated the existence of bias, i.e. that the proportion of positives differed between tests, and in these cases we calculated an additional measurement of agreement, the prevalence and bias adjusted kappa (PABAK). PABAK considers the described bias and also the underlying prevalence, which affects the (unadjusted) kappa statistic. The PABAK is calculated as 2*observed agreement-1 [34].

The agreement between the actual log titres of two serological tests was assessed using a weighted kappa. Thereby a pair of log titres with titre values closer to each other was considered to be more in partial agreement than a pair of log titres, with titre values further apart [33]. Confidence intervals for the kappa statistic of the dichotomised test results were calculated using the analytic method [35] and the bootstrap method with 100 bootstrap replication was used to calculate the confidence intervals for the weighted kappa agreement of the log titers [36,37].

The kappa values were interpreted as the following: ≤ 0 poor agreement, 0.01-0.20 slight agreement, 0.21-0.40 fair agreement, 0.41-0.60 moderate agreement, 0.61-0.80 substantial agreement and 0.81-1.0 almost perfect agreement [38]. All calculations were conducted in STATA 11.0 (Stata Corporation, 1985–2009, College Station, Texas).

## Results

### Experimental study

Not all ducks became antibody positive following the inoculation of H5N1 virus, as determined by the three serological tests (Table 1). The results of HI tests, using either chicken or horse RBCs, showed that only 8 of 10 ducks became antibody positive over the 34-day post-inoculation period, while the VN test showed 9 of 10 ducks became antibody positive. At the earliest sampling (8 dpi), the proportion of antibody-positive ducks by HI-HRBC (0.70, 95%-CI 0.35-0.93) was greater than the proportions of antibody-positive ducks by either HI-CRBC (0.40, 95%-CI 0.12-0.74) or VN (0.20, 95%-CI 0.03-0.56). All ducks testing positive at 8 dpi remained positive at the later sampling times. At 15 dpi, all three serology tests showed an identical proportion of ducks being H5 antibody-positive (0.80, 95%-CI 0.44-0.98). Although at the end of the experiment (34 dpi) the results of both HI tests revealed similar proportions of antibody-positive ducks (0.70, 95%-CI 0.35-0.93), a higher proportion of antibody-positive ducks was observed in the VN test (0.90, 95%-CI 0.55-1.00). All serum samples tested positive in the test with the least number of positives at any sampling time, were also positive by the other two tests at the same sampling time; for example, two sera that were positive at 8 dpi in the VN test were also positive in both HI tests at the same sampling. Over the entire sampling period, the HI-HRBC test detected only slightly more ducks as being antibody-positive (0.60, 95%-CI 0.33-0.87) than either the HI-CRBC test (0.57, 95%-CI 0.33-0.81) or the VN test (0.58, 95%-CI 0.40-0.76) (Table 1).

**Table 1:** Number and proportion of serum samples testing H5 antibody positive in three serological tests in ducks experimentally infected with H5N1

**Test results**	**Pre-inoculation**	**8 dpi**	**15 dpi**	**22 dpi**	**29 dpi**	**34 dpi**	**Overall**
N HI-CRBC pos	0	4	8	8	7	7	**34**
Prop HI-CRBC pos	0	0.4	0.8	0.8	0.7	0.7	**0.57**
(95%-CI)^1,2^	(0–0.31)	(0.12-0.74)	(0.44-0.98)	(0.44-0.98)	(0.35-0.93)	(0.35-0.93)	**(0.33-0.81)**
N HI-HRBC pos	0	7	8	7	7	7	**36**
Prop HI-HRBC pos	0	0.7	0.8	0.7	0.7	0.7	**0.60**
(95%-CI)^1,2^	(0–0.31)	(0.35-0.93)	(0.44-0.98)	(0.35-0.93)	(0.35-0.93)	(0.35-0.93)	**(0.33-0.87)**
N VN pos	0	2	8	8	8	9	**35**
Prop VN pos	0	0.2	0.8	0.8	0.8	0.9	**0.58**
(95%-CI)^1,2^	(0–0.31)	(0.03-0.56)	(0.44-0.98)	(0.44-0.98)	(0.44-0.98)	(0.55-1.00)	**(0.40-0.76)**

A titre cut-off of ≥ 4 log2 (16) was used to distinguish between positive and negative results. Ten ducks were sampled before inoculation and at 8, 15, 22, 29 and 34 days after virus inoculation. Abbreviations: HI-CRBC (Haemagglutination inhibition test using chicken red blood cells), HI-HRBC (Haemagglutination inhibition test using horse red blood cells), VN (Virus neutralisation test), N (number), Prop (proportion), dpi (day post inoculation), CI (confidence interval).

^1^Binominal exact confidence intervals were calculated for the proportion of positives at each sampling period.

^2^Confidence intervals for the overall proportion of positives were adjusted for a clustering of birds by sampling period (10 clusters).

The McNemar test was not significant at p < 0.05 for comparisons of the proportion of positives between two serological tests at any sampling period (except at 8 dpi between HI-HRBC and VN) or overall. Therefore, the kappa statistic was appropriate to evaluate the agreement of test results in the experimental study. Overall agreements between the two HI tests were greater than between each HI test and the VN test (Table 2). The kappa statistics for the overall dichotomised results between the HI tests indicated almost perfect agreement (κ = 0.86, 95%-CI 0.73-0.99), while substantial agreements were seen in both kappa statistics between HI-CRBC and VN (κ = 0.76, 95%-CI 0.60-0.93) and between HI-HRBC and VN (κ = 0.69, 95%-CI 0.50-0.88). Better agreement was also demonstrated for the overall weighted kappa values for the comparison of actual log titres between HI tests than the overall weighted kappa values between each HI and the VN test. When HI tests were compared at different sampling intervals, reduced agreements for dichotomised results (κ = 0.44, 0.55, and 0.19) and for the log titre results (weighted κ = 0.56, 0.49, and 0.19) were observed for the test comparisons at the beginning of the trial (8 dpi) (Table 2). However, better agreements were found between the HI tests results for the sera collected at the later samplings at 15, 22, 29 and 34 dpi.

**Table 2:** Agreement between three serological tests used for the detection of H5 antibodies in ducks experimentally infected with H5N1

**Test comparison**	**8 dpi**	**15 dpi**	**22 dpi**	**29 dpi**	**34 dpi**	**Overall Kappa**
**HI-CRBC and HI-HRBC:**						
Kappa for dichotomised results (95%-CI)^1^	0.44 (0.01-0.88)	1 (1.00-1.00)	0.74 (0.27-1.00)	1 (1.00-1.00)	1 (1.00-1.00)	**0.86 (0.73-0.99)**
Weighted kappa for log titres (95%-CI)^2^	0.56 (0.30-0.83)	0.85 (0.65-1.00)	0.61 (0.32-0.77)	0.85 (0.70-1.00)	0.66 (0.49-0.84)	**0.8 (0.71-0.85)**
**HI-CRBC and VN:**						
Kappa for dichotomised results (95%-CI)^1^	0.55 (0.04-1.00)	1 (1.00-1.00)	0.38 (−0.33-1.00)	0.74 (0.27-1.00)	0.41 (−0.18-1.00)	**0.76 (0.60-0.93)**
Weighted kappa for log titres (95%-CI)^2^	0.49 (0.27-0.71)	0.76 (0.46-0.95)	0.50 (0.25-0.66)	0.36 (0.02-0.52)	0.41 (0.24-0.65)	**0.68 (0.58-0.74)**
**HI-HRBC and VN:**						
Kappa for dichotomised results (95%-CI)^1^	0.19 (−0.10-0.49)	1 (1.00-1.00)	0.74 (0.27-1.00)	0.74 (0.27-1.00)	0.41 (−0.18-1.00)	**0.69 (0.50-0.88)**
Weighted kappa for log titres (95%-CI)^2^	0.19 (0.01-0.46)	0.71 (0.41-0.93)	0.41 (0.12-0.61)	0.37 (0.17-0.58)	0.36 (0.12-0.63)	**0.55 (0.42-0.63)**

Ten ducks were sampled before inoculation with H5N1 virus and at 8, 15, 22, 29 and 34 days post inoculation (dpi). The kappa statistic (95% confidence interval^1^) was used to compare dichotomised test results (positive versus negative) and a weighted kappa statistic (95% confidence interval^2^) was used to compare log titers at each sampling period and across all sampling periods.

^1^The analytical method was used to calculate confidence intervals for dichotomised test results [35].

^2^The bootstrap method with 100 bootstrap replications was used to calculate confidence intervals for the log titers [36,37].

Abbreviations: HI-CRBC (Haemagglutination inhibition test using chicken red blood cells), HI-HRBC (Haemagglutination inhibition test using horse red blood cells), VN (Virus neutralisation test), CI (confidence interval).

We tried to identify at what titres and at which days after inoculation discrepancies between test results were found. Discrepancies in the actual log titres for the same sera tested with different HI tests were observed at low and high log HI-CRBC titres (Table 3). For example, three duck sera showed a positive titre (5 log2) in the HI-HRBC, but were all negative (< 4 log2) when tested using the HI-CRBC. For HI-CRBC, only 40-50% of serum samples with titres of 7 log2 and 6 log2 had matching HI-HRBC titres (Table 3).

**Table 3:** Matrix indicating the number and percentage of log antibody titres obtained in two HI tests after experimental infection of ducks with H5N1

	**HI-HRBC log titres**								
**HI CRBC log titres**	**<2**	**2**	**3**	**4**	**5**	**6**	**7**	**8**	**N Total (%)**
**<2**	16	0	0		1	0	0	0	15 (28.3)
**2**	0	0	2	0	1	0	0	0	4 (5.0)
**3**	0	2	3	0	1	0	0	0	6 (10.0)
**4**	0	0	1	0	0	0	0	0	1 (1.7)
**5**	0	0	0	1	2	0	0	0	3 (5.0)
**6**	0	0	0	0	3	7	4	0	14 (23.3)
**7**	0	0	0	0	1	5	4	0	10 (16.7)
**8**	0	0	0	0	0	0	4	2	6 (10.0)
**N Total (%)**	16 (26.7)	2 (3.3)	6 (10.0)	1 (1.7)	9 (15.0)	12 (20.0)	12 (20.0)	2 (3.3)	60

Ten ducks were inoculated with H5N1 virus and were sampled before inoculation and at 8, 15, 22, 29 and 34 days post inoculation. HI titers are expressed as the log2 value of the reciprocal of the highest dilution causing inhibition of haemagglutination. The lowest detectable antibody titre was 2 log2; thus, any sera with titres below this were classified as<2 log2. HI titres of =4 log2 were classified as positive. Abbreviations: HI-CRBC (Haemagglutination inhibition test using chicken red blood cells), HI-HRBC (Haemagglutination inhibition test using horse red blood cells), N (number).

### Field study

Although the experimental study provided a complete dataset for the assessment of HI titers from individual ducks monitored over six sampling periods, the birds in that study were knowingly infected with H5 subtype virus. In order to assess sera of unknown infection status, we conducted a comparison of HI tests with sera from farmed ducks in Indonesia. Due to the limited quantity of some of the field sera, only the two HI tests (HI-HRBC and HI-CRBC) could be performed on these samples.

When the HI test results were dichotomised, more duck sera were detected as antibody-positive using HI-HRBC (41 of 518) than using the HI-CRBC test (11 of 518); and no samples that were positive with HI-CRBC were negative with HI-HRBC (Table 4). Thus, the proportion of ducks being positive was higher when the HI-HRBC test was used (0.08, 95%-CI 0.03-0.12) than when the HI-CRBC was used (0.02, 95%-CI 0.00-0.04) (McNemar test: Chi^2^ = 30.0, P < 0.001) (Table 5). Hence, we calculated in addition to the unadjusted kappa, the PABAK to evaluate the agreements between the two HI tests. Using PABAK almost perfect levels of test agreement between the two HI tests were indicated for the dichotomised results (0.88) and for the log2 titres (0.94), while the unadjusted kappa indicated only fair (0.40) to moderate (0.53) agreements (Table 5). Discrepancies were also found in the actual titre values in the same sera tested with different HI tests. Some sera had high titres of 6 or 8 log2 by HI-HRBC, but showed low titres or were negative (< 4 log2) by HI-CRBC (Table 6). Overall, when bias and prevalence adjustments were considered, agreements between tests were similar to those yielded from the experimental study.

**Table 4:** Dichotomised results for two HI test used for the detection of H5 antibodies in ducks naturally exposed to H5 avian influenza virus

	**HI-HRBC results**		
**HI-CRBC results**	**Positive**	**Negative**	**Total**
Positive	11	0	11
Negative	30	477	507
Total	41	477	518

Ducks were sampled in December 2008 in Central Java, Indonesia. A cut-off titre of ≥ 4 log2 (16) was used to distinguish between positive and negative results. Abbreviations: HI-CRBC (Haemagglutination inhibition test using chicken red blood cells), HI-HRBC (Haemagglutination inhibition test using horse red blood cells).

**Table 5:** Proportion of serum samples testing antibody positive and agreement between two HI tests in ducks naturally exposed to H5 avian influenza virus

	**HI-CRBC**	**HI-HRBC**
Proportion positive (95%-CI)^1^	0.02 (0.0-0.04)	0.08 (0.03-0.13)
Dichotimized results:		
Kappa (95%-CI)^2^	0.40 (0.24-0.57)	
PABAK	0.88	
Log titers:		
Weigthed Kappa (95%-CI)^3^	0.53 (0.45-0.62)	
PABAK	0.94	

A total of 518 ducks were sampled in December 2008. The kappa statistic (95% confidence interval^1^) was used to compare dichotomised test results (positive versus negative) and a weighted kappa statistic (95% confidence interval^2^) was used to compare log titers.

^1^Confidence intervals for the proportion of positives were adjusted for clustering of ducks by farm (54 clusters).

^2^The analytical method was used to calculate confidence intervals for dichotomised test results [35].

^3^The bootstrap method with 100 bootstrap replications was used to calculate confidence intervals for the log titers [36,37].

Abbreviations: HI-CRBC (Haemagglutination inhibition test using chicken red blood cells), HI-HRBC (Haemagglutination inhibition test using horse red blood cells), CI (confidence interval), PABAK (Prevalence and bias adjusted kappa).

**Table 6:** Matrix indicating the number and percentage of log antibody titers obtained in two HI tests in ducks naturally exposed to H5 avian influenza virus

	**HI HRBC log titres**								
**HI CRBC log titres**	**<2**	**2**	**3**	**4**	**5**	**6**	**7**	**8**	**N Total (%)**
**<2**	469	0	5	5	1	0	0	0	485 (93.6)
**2**	1	1	1	2	0	0		0	0	8 (1.5)
**3**	0	0	0	7	5	0		1	0	14 (2.7)
**4**	0	0	0	4	4	1		0	0	9 (1.7)
**5**	0	0	0	0	0	1	0	0	1 (0.2)	
**6**	0	0	0	0	0	1	0	0	1 (0.2)	
**7**	0	0	0	0	0	0	0	0	0 (0.0)	
**8**	0	0	0	0	0	0	0	0	0 (0.0)	
**N Total (%)**	470 (90.7)	1 (0.2)	6 (1.2)	9 (1.7)	18 (3.5)	10 (1.9)	3 (0.6)		1 (0.2)	518

Ducks were sampled in December 2008 in Central Java, Indonesia. A/chicken/Legok/2003(H5N1) antigen was used in both tests. HI titers are expressed as the log2 value of the reciprocal of the highest dilution causing inhibition of haemagglutination. The lowest detectable antibody titre was 2 log2; thus, any sera with titres below this were classified as < 2 log2. Abbreviations: HI-CRBC (Haemagglutination inhibition test using chicken red blood cells), HI-HRBC (Haemagglutination inhibition test using horse red blood cells), N (Number).

## Discussion

This study presents the first report of employing horse RBCs in the HI test for detecting antibodies against H5 subtype of AI virus in ducks, the species that is considered to play a major role in the maintenance of HPAI H5N1 virus in Asia [21-23]. In both the experimental and field studies, the HI-HRBC test detected a greater number of sera as positive (titre ≥ 4 log2) than the HI-CRBC test, but overall there was a very good agreement between the HI tests. Previous studies have demonstrated that performing HI tests using horse RBCs could increase the detection of HI antibodies against avian influenza viruses in human sera [13,14,17-19]. Antibodies to the viral HA glycoprotein inhibit binding of avian-derived influenza viruses to their specific sialic acid receptor, which has a sialic acid, *N*-acetylneuraminic acid α-2,3-galactose (SAα2,3Gal) linkage [19]. Horse RBCs express predominantly SAα2,3Gal linkages, whereas chicken RBCs express a mixture of SAα2,3Gal and SAα2,6Gal linkages [39], which accounts for the higher sensitivity of the avian influenza HI test using horse RBCs compared to chicken RBCs.

The experimental study showed that the HI-HRBC test was able to detect H5 antibodies at an earlier stage of serological response (8 dpi) than the HI-CRBC. At the later stages of infection, both HI-CRBC and HI-HRBC showed an equal dichotomised result. Better agreements were found between the two HI tests than between each HI test and the VN test, which might be due to differences in the binding specificities of antibodies detected in each of the tests. HI tests detect antibodies that inhibit viral hemagglutination, whereas the VN test detects antibodies that neutralize the virus and prevent replication in living cells [16]; not all antibody specificities that inhibit hemagglutination necessarily neutralize the virus, and conversely, not all virus neutralizing antibodies can inhibit hemagglutination caused by the virus [40].

The experimental study showed that not all inoculated ducks developed either HI or neutralizing antibodies by the end of the trial at 34 dpi. The virus may have failed to successfully replicate at the entry point in eyes, nasal or oral cavity of these antibody-negative ducks. Alternatively, the virus may have replicated to some degree at these mucosal entries, but was unable or had no chance to stimulate a humoral response because of a strong innate immune responses such as a rapid apoptosis mechanism [41] or RIG-I pathways [42]. Another possibility is that these antibody-negative ducks developed low HI and neutralizing antibody titres below the cut-off titre. The OIE recommended cut-off titre of 4 log2 [28] was used in this study for the dichotomization of test results. A lower cut-off of 3 log2 would have resulted in nine ducks classified seropositive by both HI tests instead of eight. Using a slightly lower HI test cut-off titre may be appropriate for the early detection of H5N1 HPAI exposure in disease free regions, but further confirmatory tests supported by epidemiological data on potential exposure status of birds are required to avoid false positive results. On the other hand, a cut-off of 4 log2 is perhaps preferable for measuring the proportion of antibody positives in countries where H5N1 HPAI is endemic and major reservoirs of all AI subtypes might exist.

A large disparity in the seroprevalence results between the two HI tests was demonstrated in the field sera. Overall, significantly (P < 0.001) more HI antibody-positive ducks were detected with HI-HRBC than HI-CRBC, but no sera that were positive in the HI-CRBC were negative in the HI-HRBC test. This indicates that the type of HI test used for assessing field sera has to be carefully chosen as the test performance will influence the reported measures of disease frequency, such as prevalence or incidence, for the population evaluated. In contrast a discrepancy between the proportions of positives identified with the HI tests was not evident in the experimental study. Compared to the experimental study, the H5N1-exposure status of ducks in the field study was unknown. Therefore, it is possible that some of the birds that tested negative in the HI-CRBC test were in the early response of H5N1 infection, which as the experimental study showed, is more likely to be detected with the HI-HRBC test than HI-CRBC.

The interpretation of the diagnostic test results should be conducted cautiously, particularly if it is performed to assess an agreement between tests using samples from different populations. The seroprevalence of ducks in the field was relatively low (8% or 2% using HI-HRBC and HI-CRBC, respectively) in contrast to the overall seroprevalence of 57-60% obtained in the experimental study. Factors related to the stage and severity of disease and the immune status vary within a population (and could be different in low- and high-prevalence populations) [43]. Therefore the distribution of individuals sampled at different stages in the infection process will influence the prevalence estimated. On the other hand, it has been highlighted that the statistical relationship between kappa and prevalence (and bias) is multifaceted and complex [33,44,45]; two tests will have a higher kappa value if the prevalence is moderate (e.g. 50%) rather than very low or very high [33]. Therefore the unadjusted kappa statistic was higher for the experimental study compared to the field study. In fact, it has been recommended by some statisticians to concentrate on populations with prevalence near 50% when comparing tests using the kappa statistic, rather than using adjusted measures such as the PABAK [45]. However, in reality we often face situations with low or high prevalence values and this was also the case in our field study. Hence, we used the PABAK to evaluate the agreement of HI tests conducted with field samples and noted a similar agreement compared to tests conducted with samples collected in the experimental study.

The variety of individuals in terms of breed and age within the population are also important factors that might influence the ability of biological assays to detect infected and uninfected animals. The experimental study used homogenous birds, while in the field study ducks of genetically diverse breeds and of different ages were sampled. In addition, given the possibility of other sub-types of AI viruses circulating on duck farms, cross re-activity could have been another factor influencing the serological test results in the field study.

Adherence to recommend guidelines, such as the OIE Manual of diagnostic tests and vaccines for terrestrial animals [28], is essential to standardize diagnostic procedures in different laboratories. For example, OIE has recommended using RBCs from specific-pathogen or specific-antibody free chickens for HI tests to detect antibodies against AI viruses, including H5N1 HPAI [28] and we did so in both our experimental and field study. However, country-specific access to specific reagents might influence laboratory specific test variability. In this regard, our study might have had some limitations related to the use of different sources of H5N1 antigen and RBCs in the serological tests of sera between the two studies. Since we used the same H5N1 virus lineage (clade 2.1.1) for antigens, with 99.9% and 99.8% homologies detected in the hemagglutinin nucleotide and amino acid sequences among both viruses respectively (only one amino acid difference was found in a residue unrelated with either antigenic or glycosylation sites) [30], we assume that the variations in the HI results are likely to be related more to the exposure status and host-specific factors of birds (e.g. age, breed) than the types of antigen used.

It was beyond the scope and objective of this study to estimate the sensitivity and specificity of the HI tests, in particular in the absence of a gold standard for detecting antibodies against avian influenza viruses. Therefore, an assessment of HI test characteristics through future studies is recommended. Overall our finding support a previous study which reported that the use of horse RBCs increased performance of HI tests in measuring AI antibodies in other bird species [20]. Thus, we suggest that the OIE should review these findings for an alternative serological method for the diagnosis and surveillance of HPAI in birds.

## Conclusion

In summary, we identified a good agreement between HI tests. However, the HI test using horse RBCs detected a higher proportion of sera as positive (titre ≥ 4 log2) than the conventional HI test based on chicken RBCs, particularly during the early response against H5N1 virus. Both HI techniques are reasonable methods for AI serosurveillance, but the HRBC-HI might be more responsive in identifying the early stage of a H5N1 HPAI serological response.

## Abbreviations

AI: Avian influenza; HPAI: Highly pathogenic AI; HI: Haemagglutination inhibition; RBC: Red blood cells; HI-HRBC: HI using horse RBCs; HI-CRBC: HI using chicken RBCs; VN: Virus neutralization; CI: Confidence interval; PABAK: Prevalence and bias adjusted kappa; κ: Kappa; dpi: Day post inoculation; PBS: Phosphate buffer saline; CPE: Cytopathic effect.

## Competing interests

The authors declare they have no competing interest.

## Authors’ contributions

HW performed the experimental study and all the serological tests. JH designed the longitudinal HPAI field study on moving ducks in Central Java, Indonesia, and conducted the statistical analysis for the results of serology tests. DEW assisted in blood and sera preparation and collected field sera. TBU advised on the HI tests with field sera. SL advised on the HI and VN tests for experimental sera and helped in interpreting the results. JB advised and helped in designing the experimental study. AJ directed the implementation of the HPAI longitudinal study. HW, JH, JB, and JM implemented and facilitated various aspects of the study. The draft of the manuscript was prepared by HW, with editorial inputs from JH, TBU, JB and JM. All authors have read and approved the final manuscript.

## References

[B1] Sims LD Ellis TM Liu KK Dyrting K Wong H Peiris M Guan Y Shortridge KE Avian influenza in Hong Kong 1997–2002 Avian Dis 2003 47 832 838 10.1637/0005-2086-47.s3.832 14575073

[B2] Sturm-Ramirez KM Ellis T Bousfield B Bissett L Dyrting K Rehg JE Poon L Guan Y Peiris M Webster RG Reemerging H5N1 influenza viruses in Hong Kong in 2002 are highly pathogenic to ducks J Virol 2004 78 4892 4901 10.1128/JVI.78.9.4892-4901.2004 15078970 PMC387679

[B3] Guan Y Smith GJ Webby R Webster RG Molecular epidemiology of H5N1 avian influenza Rev Sci Tech 2009 28 39 47 19618617 10.20506/rst.28.1.1868

[B4] Smith GJ Naipospos TS Nguyen TD de Jong MD Vijaykrishna D Usman TB Hassan SS Nguyen TV Dao TV Bui NA Evolution and adaptation of H5N1 influenza virus in avian and human hosts in Indonesia and Vietnam Virology 2006 350 258 268 10.1016/j.virol.2006.03.048 16713612

[B5] Alexander DJ Summary of avian influenza activity in Europe, Asia, Africa, and Australasia, 2002–2006 Avian Dis 2007 51 161 166 10.1637/7602-041306R.1 17494548

[B6] Wang G Zhan D Li L Lei F Liu B Liu D Xiao H Feng Y Li J Yang B H5N1 avian influenza re-emergence of Lake Qinghai: phylogenetic and antigenic analyses of the newly isolated viruses and roles of migratory birds in virus circulation J Gen Virol 2008 89 697 702 10.1099/vir.0.83419-0 18272760 PMC2885753

[B7] Amonsin A Lapkuntod J Suwannakarn K Kitikoon P Suradhat S Tantilertcharoen R Boonyapisitsopa S Bunpapong N Wongphatcharachai M Wisedchanwet T Genetic characterization of reassortant influenza A virus (H5N1), Thailand Virol J 2010 7 233 10.1186/1743-422X-7-233 20843374 PMC2949837

[B8] Wan XF Nguyen T Davis CT Smith CB Zhao ZM Carrel M Inui K Do HT Mai DT Jadhao S Evolution of highly pathogenic H5N1 avian influenza viruses in Vietnam between 2001 and 2007 PLoS One 2008 3 e3462 10.1371/journal.pone.0003462 18941631 PMC2565130

[B9] Chakrabarti AK Pawar SD Cherian SS Koratkar SS Jadhav SM Pal B Raut S Thite V Kode SS Keng SS Characterization of the Influenza A H5N1 Viruses of the 2008–09 Outbreaks in India Reveals a Third Introduction and Possible Endemicity PLoS One 2009 4 e7846 10.1371/journal.pone.0007846 19924254 PMC2775943

[B10] Ahmed SSU Ersboll AK Biswas PK Christensen JP The space-time clustering of highly pathogenic avian influenza (HPAI) H5N1 outbreaks in Bangladesh Epidemiol Infect 2010 138 843 852 10.1017/S0950268810000178 20109257

[B11] Hirst GK The quantitative determination of influenza virus and antibodies by means of red cell agglutination J Exp Med 1942 75 49 64 10.1084/jem.75.1.49 19871167 PMC2135212

[B12] Pedersen JC Spackman E Hemagglutination-inhibition test for avian influenza virus subtype identification and the detection and quantitation of serum antibodies to the avian Influenza virus Avian Influenza Virus 2008 Totowa, NJ: Humana Press 53 66 10.1007/978-1-59745-279-3_8 18370041

[B13] Noah DL Hill H Hines D White EL Wolff MC Qualification of the hemagglutination inhibition assay in support of pandemic influenza vaccine licensure Clin Vaccine Immunol 2009 16 558 566 10.1128/CVI.00368-08 19225073 PMC2668270

[B14] Kayali G Setterquist SF Capuano AW Myers KP Gill JS Gray GC Testing human sera for antibodies against avian influenza viruses: horse RBC hemagglutination inhibition vs. microneutralization assays J Clin Virol 2008 43 73 78 10.1016/j.jcv.2008.04.013 18571465 PMC2574547

[B15] Rowe T Abernathy RA Hu-Primmer J Thompson WW Lu X Lim W Fukuda K Cox NJ Katz JM Detection of antibody to avian influenza A (H5N1) virus in human serum by using a combination of serologic assays J Clin Microbiol 1999 37 937 943 10074505 10.1128/jcm.37.4.937-943.1999 PMC88628

[B16] Bachmann MF Ecabert B Kopf M Influenza virus: a novel method to assess viral and neutralizing antibody titers in vitro J Immunol Methods 1999 225 105 111 10.1016/S0022-1759(99)00034-4 10365787

[B17] Louisirirotchanakul S Lerdsamran H Wiriyarat W Sangsiriwut K Chaichoune K Pooruk P Songserm T Kitphati R Sawanpanyalert P Komoltri C Erythrocyte binding preference of avian influenza H5N1 viruses J Clin Microbiol 2007 45 2284 2286 10.1128/JCM.00921-07 17522271 PMC1933005

[B18] Stephenson I Wood JM Nicholson KG Charlett A Zambon MC Detection of anti-H5 responses in human sera by HI using horse erythrocytes following MF59-adjuvanted influenza A/Duck/Singapore/97 vaccine Virus Res 2004 103 91 95 10.1016/j.virusres.2004.02.019 15163495

[B19] Stephenson I Wood JM Nicholson KG Zambon MC Sialic acid receptor specificity on erythrocytes affects detection of antibody to avian influenza haemagglutinin J Med Virol 2003 70 391 398 10.1002/jmv.10408 12767002

[B20] Humberd J Guan Y Webster RG Comparison of the replication of influenza A viruses in Chinese ring-necked pheasants and chukar partridges J Virol 2006 80 2151 2161 10.1128/JVI.80.5.2151-2161.2006 16474123 PMC1395373

[B21] Hulse-Post DJ Sturm-Ramirez KM Humberd J Seiler P Govorkova EA Krauss S Scholtissek C Puthavathana P Buranathai C Nguyen TD Role of domestic ducks in the propagation and biological evolution of highly pathogenic H5N1 influenza viruses in Asia Proc Natl Acad Sci U S A 2005 102 10682 10687 10.1073/pnas.0504662102 16030144 PMC1180796

[B22] Gilbert M Xiao X Pfeiffer DU Epprecht M Boles S Czarnecki C Chaitaweesub P Kalpravidh W Minh PQ Otte MJ Mapping H5N1 highly pathogenic avian influenza risk in Southeast Asia Proc Natl Acad Sci U S A 2008 105 4769 4774 10.1073/pnas.0710581105 18362346 PMC2290786

[B23] Sturm-Ramirez KM Hulse-Post DJ Govorkova EA Humberd J Seiler P Puthavathana P Buranathai C Nguyen TD Chaisingh A Long HT Are ducks contributing to the endemicity of highly pathogenic H5N1 influenza virus in Asia? J Virol 2005 79 11269 11279 10.1128/JVI.79.17.11269-11279.2005 16103179 PMC1193583

[B24] Kida H Yanagawa R Matsuoka Y Duck influenza lacking evidence of disease signs and immune response Infect Immun 1980 30 547 553 7439994 10.1128/iai.30.2.547-553.1980 PMC551346

[B25] Toth TE Norcross NL Precipitating and agglutinating activity in duck anti-soluble protein immune sera Avian Dis 1981 25 338 352 10.2307/1589927 6167255

[B26] Henning J Henning K Vu LT Yulianto D Meers J The role of moving duck flocks in the spread of Highly Pathogenic Avian Influenza (HPAI) virus in Vietnam and Indonesia Proceedings of the International Society for Veterinary Epidemiology and Economics (ISVEE) XII, Theme 2 - Investigation of determinants and distribution of disease: Avian influenza, Disease distribution & determinants: 9-14 August 2009, Durban, South Africa 2009 30

[B27] Henning J Wibawa H Morton J Usman TB Junaidi A Meers J Scavenging ducks and transmission of highly pathogenic avian influenza, Java, Indonesia Emerg Infect Dis 2010 16 1244 1250 10.3201/eid1608.091540 20678318 PMC3298304

[B28] OIE Chapter 2.3.4. Avian Influenza (Version adopted in May 2009) Manual of diagnostic tests and vaccines for terrestrial animals 2011 2011 http://www.oie.int/fileadmin/Home/eng/Health_standards/tahm/2.03.04_AI.pdf.

[B29] WHO WHO manual on animal influenza diagnosis and surveillance 2002 http://www.who.int/csr/resources/publications/influenza/whocdscsrncs20025rev.pdf.

[B30] Wibawa H Henning J Wong F Selleck P Junaidi A Bingham J Daniels P Meers J A molecular and antigenic survey of H5N1 highly pathogenic avian influenza virus isolates from smallholder duck farms in Central Java, Indonesia during 2007–2008 Virol J 2011 8 425 10.1186/1743-422X-8-425 21896207 PMC3179459

[B31] Anonymous Stata Users’s Guide Release 11 2009 College Station, Texas: Stata Press 262 273

[B32] Anonymous Stata Users’s Guide Release 11 2009 College Station, Texas: Stata Press 1420 1424

[B33] Dohoo I Martin W Stryhn H Veterinary Epidemiologic Research 2009 2 Charlottetown, Prince Edward Island, Canada: AVC Inc

[B34] Byrt T Bishop J Carlin JB Bias, prevalence and kappa J Clin Epidemiol 1993 46 423 429 10.1016/0895-4356(93)90018-V 8501467

[B35] Fleiss JL Levin B Paik MC Statistical Methods for Rates and Proportions 2003 3 New York: Wiley

[B36] Efron B Tibshirani R An Introduction to the Bootstrap 1993 London: Chapman & Hall

[B37] Lee J Fung KP Confidence interval of the kappa coefficient by bootstrap resampling Psychiatry Res 1993 49 97 98 10.1016/0165-1781(93)90033-D 8140185

[B38] Landis JR Koch GG Measurement of Observer Agreement for Categorical Data Biometrics 1977 33 159 174 10.2307/2529310 843571

[B39] Ito T Suzuki Y Mitnaul L Vines A Kida H Kawaoka Y Receptor specificity of influenza A viruses correlates with the agglutination of erythrocytes from different animal species Virology 1997 227 493 499 10.1006/viro.1996.8323 9018149

[B40] Gerhard W Mozdzanowska K Furchner M Washko G Maiese K Role of the B-cell response in recovery of mice from primary influenza virus infection Immunol Rev 1997 159 95 103 10.1111/j.1600-065X.1997.tb01009.x 9416505

[B41] Kuchipudi SV Dunham SP Nelli R White GA Coward VJ Slomka MJ Brown IH Chang KC Rapid death of duck cells infected with influenza: a potential mechanism for host resistance to H5N1 Immunol Cell Biol 2012 90 116 123 10.1038/icb.2011.17 21423263 PMC3257048

[B42] Barber MR Aldridge JR Jr Webster RG Magor KE Association of RIG-I with innate immunity of ducks to influenza Proc Natl Acad Sci U S A 2010 107 5913 5918 10.1073/pnas.1001755107 20308570 PMC2851864

[B43] Greiner M Gardner IA Epidemiologic issues in the validation of veterinary diagnostic tests Prev Vet Med 2000 45 3 22 10.1016/S0167-5877(00)00114-8 10802331

[B44] Vach W The dependence of Cohen's kappa on the prevalence does not matter J Clin Epidemiol 2005 58 655 661 10.1016/j.jclinepi.2004.02.021 15939215

[B45] Hoehler FK Bias and prevalence effects on kappa viewed in terms of sensitivity and specificity J Clin Epidemiol 2000 53 499 503 10.1016/S0895-4356(99)00174-2 10812322

